# Impacts of Quarterly Sow Mass Vaccination with a Porcine Reproductive and Respiratory Syndrome Virus Type 1 (PRRSV-1) Modified Live Vaccine in Two Herds

**DOI:** 10.3390/vaccines9101057

**Published:** 2021-09-23

**Authors:** Kasper Pedersen, Charlotte Sonne Kristensen, Lise Kirstine Kvisgaard, Lars Erik Larsen

**Affiliations:** 1 SEGES Danish Pig Research Centre, Agro Food Park 15V, 8200 Aarhus N, Denmark; KAPE@seges.dk (K.P.); CSK@seges.dk (C.S.K.); 2 Faculty of Health and Medical Sciences, Department of Veterinary and Animal Sciences, University of Copenhagen, 1870 Frederiksberg C, Denmark; likik@sund.ku.dk

**Keywords:** PRRSV, mass vaccination, serological assay, immune response

## Abstract

In recent years, there has been a considerable increase in the use of Modified Live PRRSV Vaccines (MLV) for mass vaccination in Denmark. The potential risks and negative impact of this strategy have been sparsely studied. The aim of this study was to investigate the impact of quarterly sow mass vaccination in two Danish sow herds. The study was performed as an observational prospective cohort of 120 sows in each of two commercial breeding herds in a paired design. Blood samples were taken from sows and oral fluid samples from nursery pigs (four to ten weeks old) before and after vaccination. The presence of PRRSV-1 RNA was measured by real time quantitative reverse transcription-polymerase chain reaction (RT-qPCR), and the level of PRRSV-1 specific antibodies was measured by two different serological assays. PRRS virus was not detected in the sow herds two days before and two weeks after vaccination, but the vaccine strain virus was detected in the nursery pigs. The prevalence of sows without antibodies towards PRRSV-1 went from 6–15% before vaccination to 1–4% after vaccination depending on the serological assay used, despite the fact that they had previously been repeatedly vaccinated. Four sows tested negative for antibodies in both assays after vaccination.

## 1. Introduction

Porcine Reproductive and Respiratory Virus (PRRSV) remains a challenge for sow herds globally. In Denmark, the use of Modified Live PRRSV Vaccines (MLV) has increased during the past three years (https://vetstat.fvst.dk/vetstat/, accessed on 5 April 2021) (supplementary material, Figure S1). A possible explanation for this increase may be an outbreak of PRRSV-1 in a boar station in 2019. Interestingly, the virus strain responsible for the outbreak was a recombination between two PRRSV modified live virus (MLV) vaccines [1].

Following an acute outbreak of PRRSV, most producers aim to establish a “positive stable” sow herd [2,3], whereby all breeding animals are immunised prior to first service followed by repeated sow mass vaccination (SMV) up to four times yearly [4,5,6]. In Denmark, 98.8 percent of the PRRSV-1 vaccines sold for breeding animals in 2020 were MLV vaccines (https://vetstat.fvst.dk/vetstat/, accessed on 5 April 2021), which is in accordance with the situation in other countries. The prescription of MLV vaccines towards reproductive failure in sow herds is supported by research in the field [7,8,9,10]. However, repeated exposure of breeding animals to PRRSV MLV is often “off-label” use and may lead to negative side effects [11,12]. Thus, the attenuated vaccine strain may persist in the animals after vaccination [13] or even revert to virulence [1]. Furthermore, vaccination of pregnant and lactating sows entails a risk of vertical or horizontal transmission to the fetus and/or piglets depending on the gestation stage and the immune status of the sows [13,14,15,16,17]. Lastly, repeated exposure to the same antigen may lead to an “anergy state”, where lymphocytes do not react to a foreign substance or if immunoglobulins have reduced affinity maturation, as indicated for human seasonal influenza vaccines [18] and in a previous preliminary study on PRRSV [15]. The potential risks of SMV have therefore raised concerns among veterinarians and scientists.

The aim of this study was to investigate the impact of SMV on viremia and antibody levels in sows before and after vaccination. Furthermore, a comparison of the results of the two most commonly used antibody assays was completed, since previous studies have revealed different performances of different tests [13].

## 2. Materials and Methods

### 2.1. Farms

The study was performed in two similar sow herds, F1 and F2, each housing approximately 2000 sows. Both herds performed simultaneous SMV with the MLV Porcilis PRRS every seventeenth week. The dam line was the DanBred Hybrid (DanBred Landrace and DanBred Yorkshire) inseminated with DanBred Duroc semen. Both herds received PRRSV-vaccinated (Porcilis PRRS, MSD Animal Health, The Netherland and Ingelvac PRRS, Boehringer Ingleheim Animal Health, Germany) gilts from the same quarantine unit. The herds weaned piglets at four weeks of age to two different nursery units (N1 and N2) alternately every week (flow diagram in Figure S2 in supplementary material). The specific pathogen free (SPF) status was Blue SPF + Mycoplasma hyopneumoniae + Actinobacillus pleuropneumoniae type 12 + PRRSV-1 + PRRSV-2 verified yearly by serological antibody detection. The farrowing unit was prepared as a sectioned unit but was continuously run with commingling of different batches of sows. Weaned pigs between the individual sow batches were transferred to so-called “baby stables” in the respective sow herd and were subsequently transferred to the nursery sites together with the next batch of weaned piglets.

### 2.2. Experimental Design

The study was designed as an observational prospective cohort study. Two groups of animals (*n* = 60) were stratified into each cohort based on the risk of shedding PRRSV-1 after vaccination, i.e., late pregnant sows around day 105 of gestation at SMV time and lactating sows just after farrowing on days 3 to 6 of lactation at SMV time. Blood samples were taken from the sows two days before intramuscular vaccination (−2DPV) with Porcilis PRRS (MSD Animal Health, the Netherlands) and again 14 days after vaccination (WPV2). At WPV2, udder wiping [19] was also performed for all sows in both groups using a cotton wipe. All blood samples were taken from the vena jugularis in BD Vacutainer^®^ serum tubes with coagulation activator Hemogard™ (Becton, Dickinson and Company, Franklin Lakes, NJ, USA).

On the same day as the blood collection, and at 12 weeks after vaccination (WPV12), oral fluid (OF) samples were collected in the nursery units. The samples were collected in four pens in each stable (of 24 pens with approx. 1200 nursery pigs), corresponding to pooled oral fluid from approximately 200 nursery pigs in each stable. Ropes were installed in each pen for 30–90 min to ensure that the pigs bit and sucked sufficiently on the rope. Afterwards, each rope was twisted and the saliva was transferred to a separate plastic container for each pen. This was placed in an ice bath in order to cool the samples down and inactivate enzyme activity [20,21].

All samples were stored at 5 °C before and during transport to the Department of Veterinary and Animal Sciences, Faculty of Health and Medical Sciences, University of Copenhagen.

### 2.3. Laboratory Analysis

All laboratory analyses were performed at the University of Copenhagen, with the exception of the MFIA, which was performed at the National Veterinary Institute, Technical University of Denmark, Lyngby, Denmark. Serum was separated by centrifugation of the blood samples at 2600× *g* for ten minutes, and total RNA extracted using the QIAcube HT robot (QIAGEN) followed by a purification step of 200 µL aliquots utilizing the protocol: “Cador Pathogen 96 QIACube HT V3”. Oral fluid samples were processed with metal bead and homogenization on a TissueLyser II at 30 Hz, 15 s, following centrifugation for 3 min at 5500× *g*. In total, 140 µL aliquots PBS-liquid was separated from the udder wipes by 15 s. vortex. Total RNA was extracted from udder samples and oral fluids with the QIAamp^®^ Viral RNA Mini Kit (QIAGEN) using the QIAGEN QIAcube Connect extraction robot and protocol: “Purification of viral RNA from cell-free body fluids”. In extractions, known PRRSV-positive and negative controls were included. The full procedure was performed as previously described [22]. All serum and udder wipe samples were analysed by RT-qPCR [23] in pools of five, and OF in pools corresponding to each stable as previously described [23]. Samples were considered positive for PRRSV-1 if the Cq-value was ≤40.0. Representative positive samples were selected for sequencing of ORF5 using the methods described previously [24,25].

Diluted (1:40) serum samples were tested for antibodies against PRRSV with the Enzyme-linked immunosorbent assay (ELISA) (IDEXX PRRS X3 Ab Test, Idexx Hoofddrop, the Netherlands) (E1) and the Multiplexed Fluorometric Immuno Assay (MFIA) (Swinecheck MP PRRSV type 1 and 2, App2, App6 and App12; Biovet; Saint-Hyacinthe, Canada) using the instructions recommended by the manufacturer but using an in-house validated cut-off at 0.25 instead of the recommended cut-off of 0.35. In MFIA, the PRRSV type was determined by calculating the ratio between S/P results of PRRSV-1 and 2 antibodies (ln(S/P% type 2/S/P% type 2)), because antibodies may cross-react with PRRSV-1 and 2 antigens. If antibodies of PRRSV-1 and 2 were present with approximately the same S/P value, the results were considered inconclusive (ratio = 0.0) and, thus, not included in further analysis. Postvaccination sera from animals testing negative in both Idexx ELISA and MFIA before and after vaccination and randomly chosen sera were submitted to a virus neutralization test (VNT) to detect other than N-protein dependent antibodies. Sera were initially heat inactivated (56 °C in 30 min) and diluted 1:2 in Minimum Essential Medium (MEM) with 1% of Penicillin-Streptomycin. Two-fold duplicates dilution series of serum to 1:2048 in 96-wells plates were made with cell media of MEM, 2% foetal bovine serum, 1% Penicillin-Streptomycin and Glutamine, and 1% of MEM Non-Essential Amino Acids. Vaccine-strain virus isolates of both PRRSV-1 and PRRSV-2 at 2 × 10^3^ TCID_50_/mL were included before a one-hour incubation period in humanized atmosphere (37 °C and 5% CO_2_). A total of 1.5 × 10^4^ MARC 145 cells/well of 15th passage were added before incubation for 72 h in humanized atmosphere. Known PRRSV-1 and PRRSV-2 positive virus neutralising sera, negative and positive virus controls were included at each plate. Fixation of cells were performed with Phosphate Buffered Saline (PBS) wash and absolute EtOH. Staining was prepared by wash with 0.1% Tween^®^ 20 in PBS (PBS-T), and PRRSV monoclonal antibody binding with SDOW 17-A in blocking (5% nonfat dry milk) buffer (1:500) for one hour in humanized atmosphere followed by PBS-T wash. Polyclonal Rabbit Anti-Mouse Ig/HRP in blocking buffer (1:200) was added before incubation in 30 min. Staining was performed by 3-Amino-9-ethylcarbazole in acetate buffer (pH 5, 1:50) and H_2_O_2_ (1:2000). Neutralisation titres of at least 1:8 were considered of biological significance.

### 2.4. Statistical Analysis

Data management was performed with Microsoft^®^ Excel Version 16.49, and statistical analysis was performed in Graph Pad Prism 9 Version 9.1.2. In all the statistical analysis, the level of statistical significance (*p*) was 5%. First, results from both serological assays (Idexx ELISA and MFIA) were analysed with a descriptive statistical method and presented in boxplots. The results were then analysed for Gaussian distribution and the variances compared between herds and before and after vaccination. The proportion of seroconverted animals on each farm before and after vaccination was compared in a one-sided Fisher’s exact test. The comparisons of the S/P ratios on each farm before and after vaccination were performed using a one-tailed Student’s t-test (F1, Idexx ELISA) and one-sided Wilcoxon matched-pairs signed rank test (F1 MFIA, F2 Idexx ELISA and MFIA). Because of non-parametric data, a Spearman’s correlation was run to determine the relationship between the increase in S/P ratio and the S/P ratio before vaccination and to determine any relationship between the Idexx ELISA and MFIA. Agreement between results in the Idexx ELISA and MFIA was compared in a dichotomous setup with GraphPad’s McNemar’s test. For the initial analysis of the mean/median between the two assays, a relative to positive value was established (e.g., result of 0.4 in Idexx ELISA corresponded to 1, and a result of 0.25 in MFIA corresponded to 1). All comparisons were subsequently non-parametric, and, therefore, a two-tailed Wilcoxon matched-pairs signed rank test was performed, except on Farm 2 before vaccination, where a two-tailed paired Student’s t-test was performed. GraphPad Software was used to calculate the agreement between all ELISAs using Cohen’s kappa coefficient (κ).

## 3. Results

### 3.1. RT-qPCR

All 480 serum samples and 240 udder wipe samples taken before and after vaccination tested negative in the RT-qPCR (data shown in supplementary material Table S1). Of the 236 OF samples from nursery pigs, 11 were positive for PRRSV (Table 1). PRRSV-1 was detected in one stable (stable 13) before vaccination (Cq = 39) in N1 and in two stables (stable 9 and 10) 12 weeks after vaccination (Cq = 34 and Cq = 33) in N2. PRRSV-2 was detected in two stables (stable 0 and 10) in N1 (Cq = 31 and Cq = 32) and in one stable (stable 3) in N2 (Cq = 32) before vaccination. Two weeks after vaccination, PRRSV-2 was detected in one stable (stable 6) in N2 (Cq = 38) and, 12 weeks after vaccination, PRRSV-2 was detected in four stable (stable 8, 13, 14 and 15) in N1 (Cq = 29–35).

### 3.2. Sequence Analysis

Two samples taken 12 weeks after vaccination that tested positive for PRRSV-1 and two samples that tested positive for PRRSV-2 were subjected to ORF5 sequence analyses. The detected PRRSV-1 shared a high level (99.67%) of genetic similarity to the Porcilis PRRS vaccine strain ‘DV’ with two nucleotides difference, where one mutation led to one amino acid change (N174S). The PRRSV-2 viruses were almost identical (98.34% and 99.00%) to the VR2332 vaccine strain (Ingelvac PRRS; Boehringer Ingelheim, Germany) with ten and six nucleotides difference in ORF5, respectively. These differences led to six (6) (C10Y, N33D, D34N, N58D, G151K, R156W) and three (3) (C10Y, D34N, N58D) amino acid substitutions compared to the VR2332 vaccine strain. The amino acid substitutions in the PRRSV-2 case samples did not alter the putative neutralizing epitope (aa37–45: SHL/FQLIYNL) [26].

### 3.3. Test for Antibodies

Descriptive statistical results from both serological tests are shown in Table 2 (and in Table S1). Due to a limited amount of serum available, five samples from Farm 1 after vaccination were not tested.

Before the first vaccination, eighteen (15.0%) and nine (7.5%) of the sows were negative for PRRSV antibodies on Farm 1 and Farm 2, respectively, when tested with the Idexx ELISA. A total of fourteen (77.8% of all seronegative) and eight (88.9% of all seronegative) sows from Farms 1 and 2, respectively, seroconverted after vaccination, and the number of PRRSV antibody negative animals was then reduced to four (3.3%) and two (1.7%), respectively. Five of the sows found seronegative after vaccination were also negative before vaccination (three second parity sows, one third parity sow and one fourth parity sow). One first parity sow on Farm 2 that was positive prior to vaccination tested negative after vaccination by the Idexx ELISA. The number of PRRSV seroconverted animals in the Idexx ELISA increased significantly on both farms (one-sided Fisher’s exact test F1: *p* < 0.01, F2: *p* < 0.05).

In the MFIA test, 19 (15.8%) and 11 (9.2%) of the sows tested negative for PRRSV-1 antibodies before vaccination on F1 and F2, respectively. Two weeks after vaccination, thirteen (68.4% of all seronegative) and nine (81.8% of seronegative) sows had seroconverted, but five sows (4.2%) on Farm 1 and one sow (0.8%) on Farm 2 were still negative for antibodies against PRRSV-1 (two first, three second and one fourth parity). Two weeks after vaccination, the proportion of PRRSV-1 positive animals increased significantly on both farms (one-sided Fisher’s exact test F1: *p* < 0.01, F2: *p* < 0.01).

Before vaccination, seven and four sows tested positive for antibodies towards PRRSV, but the typing was inconclusive (ratio = 0.0). Two weeks after vaccination, the number of sows with inconclusive typing had increased to ten and eleven for each farm, respectively.

**Figure 1:**
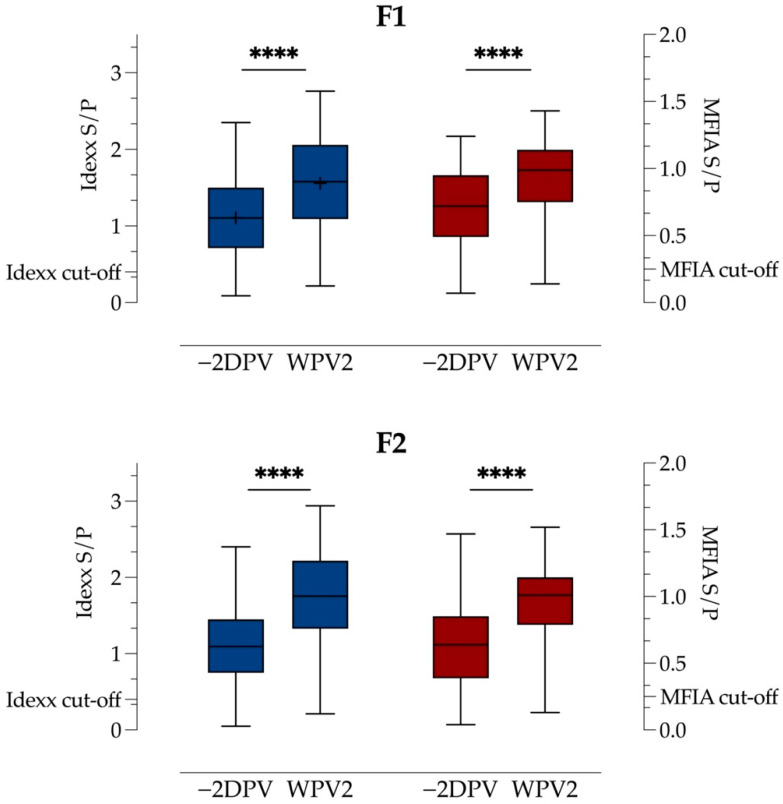
Boxplots showing S/P results of Idexx ELISA (blue) on the first *y*-axis and MFIA (red) on the second *y*-axis for Farms 1 (F1) and 2 (F2), before (−2DPV) and after (WPV2) mass vaccination with a PRRSV-1 MLV vaccine as minimum, median and maximum as well as quartiles of 25, 50, 75. The cut-off for each assay is given on each axis. “+” indicates mean. **** is *p* < 0.0001 as result of the comparison of results −2DPV and WPV2for each analysis.

The quantity of antibodies in each test (Idexx ELISA or MFIA) for Farms 1 and 2 are presented in Figure 1. In the Idexx ELISA, the mean S/P ratio before vaccination was 1.11 and the median was 1.10 for Farms 1 and 2, respectively. There was a significant increase to 1.56 on Farm 1 (*p* < 0.0001, *M*: 0.45, *SD*: 0.46, 95% CI 0.37–0.54) and 1.76 on Farm 2 (*p* < 0.0001, median: 0.60, 95% CI 0.48–0.69) two weeks after vaccination. In the MFIA, the median S/P ratios were 0.64 and 0.60 prior to vaccination, which increased significantly (*p* < 0.0001, median: 0.23, 95% CI 0.21–0.32 and *p* < 0.0001, median: 0.31, 95% CI 0.27–0.39) to 0.96 and 1.02 after vaccination on Farms 1 and 2, respectively.

**Figure 2:**
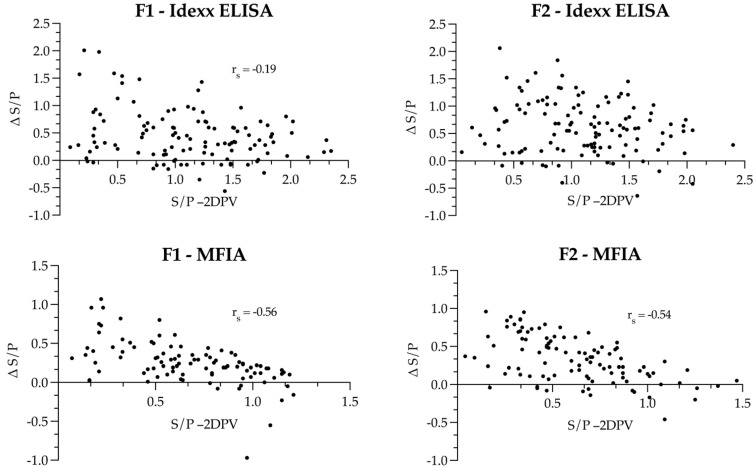
Scatterplots with difference in S/P ratio before and after mass vaccination (Δ S/P) plotted against the S/P ratio before vaccination (−2DPV) of both Idexx ELISA and MFIA(−2DPV) on Farm 1 (F1) and Farm 2 (F2). Correlation coefficients for the monotonic relationship are given as *rs* in each figure.

There was a very weak negative monotonic relationship between S/P values before vaccination and the difference in the S/P values two weeks after vaccination in the Idexx ELISA results of Farm 1 (*r_s_* = −0.19, *p* < 0.05, 95% CI −0.36–0.00), whereas no relationship was detected (*p* > 0.05) on Farm 2. In the MFIA, the correlation seemed to be moderate with *r_s_* = 0.56 and *r_s_* = −0.54 on Farms 1 (*p* < 0.0001, 95% CI −0.68–0.40) and 2 (*p* < 0.0001, 95% CI −0.67–0.38), respectively (Figure 2).

Postvaccination sera from three Idexx ELISA- and MFIA-negative animals and from four randomly chosen antibody-positive animals in each herd (*n* = 11) were analysed in the VNT. Of antibody-positive animals, seven yielded positive neutralization of PRRSV-1, and sera from three of eight animals did not neutralize PRRSV-2. Sera from one of the three Idexx ELISA- and MFIA-negative animals managed to prevent both PRRSV-1- and 2-infection of MARC 145 cells at 1:32 and 1:8 dilutions, respectively.

### 3.4. Agreement in Results between the Idexx ELISA and the MFIA Serological Tests

In a dichotomous result comparison (Table 3 and Table S2), no statistically significant difference (McNemar’s test *p* > 0,05) was detected either in the individual farm or in total, as well as before or after vaccination or in total.

**Figure 3:**
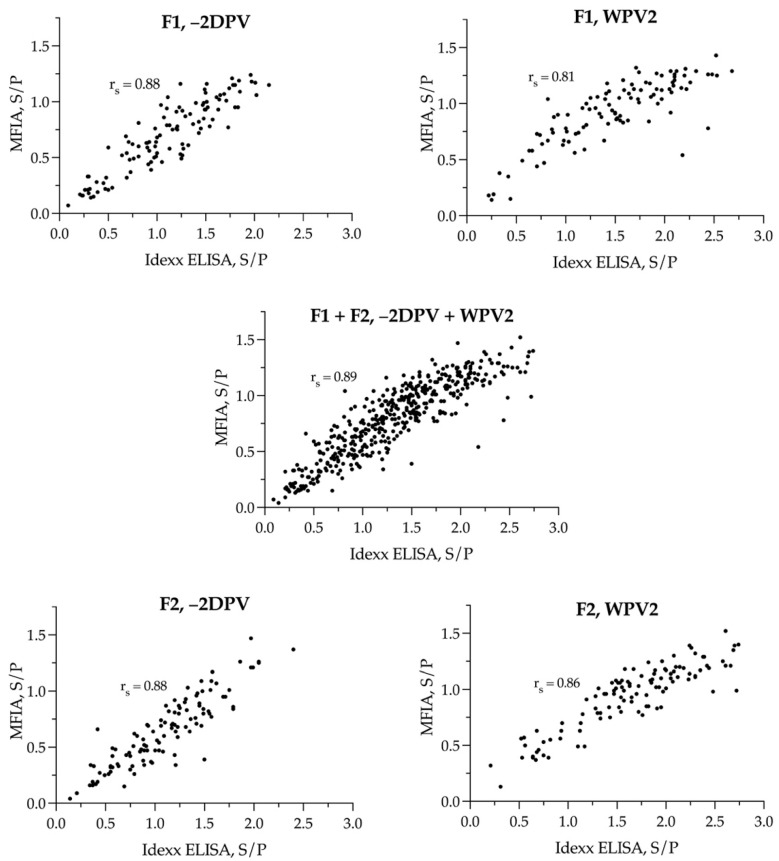
Scatterplots with PRRSV-1 S/P values as results of both Idexx ELISA (*x*-axis) and MFIA (*y*-axis) before (−2DPV) and after PRRSV-1 MLV mass vaccination (WPV2) on Farm 1 (F1) and Farm 2 (F2), and in total for both herds (Both) in the study. Correlation coefficients for the monotonic relationship are given as *rs* in each figure.

On Farm 1, there was no difference in S/P ratios between the median results of the two tests prior to vaccination (*p* > 0.05) and two weeks after (*p* > 0.05). On Farm 2, there was a significant difference between the mean S/P ratios prior to vaccination (*p* < 0.001, *M* = −0.17, *SD*: 0.55, 95% CI −0.27–0.06) and between medians two weeks after vaccination (*p* < 0.0001, median = −0.27, 95% CI −0.48–0.20). A Spearman’s correlation was subsequently run to determine the relationship between S/P results of the two serological assays (Figure 3, supplementary material Table S3). There was a statistically significant monotonic association between results in the Idexx ELISA and MFIA in all comparisons (*p* < 0.0001) (supplementary material S3). All correlation coefficients (*rs*) revealed a positive relationship between the Idexx ELISA and MFIA. They were 0.88 on both farms prior to vaccination, 0.81 two weeks after vaccination on Farm 1 and 0.86 after vaccination on Farm 2. For all samples combined, the correlation coefficient was 0.89. The results of the Cohen’s kappa agreement, combined for both herds −2DPV and WPV2, are presented in Table 4 (and for each herd in supplementary material Table S4). Overall, in both herds it ranged from 0.65 to 0.77.

## 4. Discussion

### 4.1. Virus

This study did not detect PRRS virus in the two sow herds two days before and two weeks after mass vaccination with Porcilis PRRS (MSD Animal Health, The Netherlands). The sampled fraction of animals corresponded to approximately six percent of the sows, which indicates that the prevalence of virus-positive animals was below 5% (95% confidence level) after vaccination. Lactating sows and sows in late gestation were selected for sampling because the development of viremia in these groups could potentially lead to severe consequences for the foetus/newborn piglets. The results are in accordance with previous findings by Leberet et al. (2021) following mass vaccination with ReproCyc^®^ PRRS EU (Boehringer Ingelheim, Ingelheim, Germany) in a positive and stable breeding herd [27]. In contrast, another study from Spain found that the risk of having an unstable herd with virus circulation was higher in herds that were vaccinated with MLV [28].

Contrary to the findings in the sow herds, the PRRSV vaccine strain was detected in nursery pigs both before and 12 weeks after mass vaccination. Therefore, although the herd fulfilled the criteria for being classified as stable negative by having seropositive and virus-negative sows and virus-negative weaned piglets [2], the nursery pigs still became PRRS virus-positive. The observation in the herds was that they failed to follow some of the basic rules for effective PRRSV control. The farrowing unit was not sectioned according to age groups, and some pigs remained in the sow herd after weaning. This herd was, therefore, at high risk of having PRRSV introduced into the sow herd, and PRRSV-1 was indeed detected in some pigs the day after weaning, half a year after the study was finalised (data not shown). It is also possible that the reason for the negative virus test of sows in our study was that the low prevalence virus circulation might not have been detected due to the limited sample size.

### 4.2. Effect of SMV

Both breeding herds included in this study were known to be infected with both types of PRRSV, which might have influenced the outcome of the study, especially in the comparisons between the two tests. However, according to the discriminatory test, MFIA, only a few sows had an S/P ratio indicating the dominance of antibodies towards PRRSV-2. Therefore, it is most likely that the presence of a low level of PRRSV-2 in the herd had a minor impact on the serological status of the sows.

The vaccination of sows that had previously been vaccinated with the same vaccine several times resulted in a significant increase in the proportion of animals with antibodies towards PRRSV-1 on both farms and in both tests. Following re-vaccination, there were 96–99% PRRSV-1 antibody-positive sows depending on the test and farm. There were, however, still four animals of different parity that tested negative in both tests two weeks after re-vaccination. One of the Idexx ELISA and MFIA-negative animals were shown to have low titres of neutralizing antibodies against PRRSV, indicating that some of the ELISA and/or MIFA negative animals may have low titres of antibodies. These results are probably due to the fact that the samples for ELISA and MIFA were diluted 40 times prior to test. Negative animals could, mistakenly, not have been vaccinated or they represent so-called non-responders [29], or it could be that the results indicate a state of anergy in a very small number of animals. A similar study (Díaz et al. (2019)) also found a few animals that tested negative for antibodies against PRRSV-1 in a range of different tests, including four ELISAs, IPMA, VNT and ELISPOT after mass vaccination [13]. The mechanism behind this is not known, although it may be due to the induction of anergy as seen in some human studies with repeated influenza virus vaccines [18,30,31] or other basic immunological features such as variability of MHC class II structures, etc. [29].

Other animals had different results in the two tests. For example, one sow on each farm had negative results both before and after vaccination in the Idexx ELISA, but was positive in the MFAI after vaccination. These results can be regarded either as false negatives or false positives depending on the unknown true status of the animals. The same is expected of two other sows on Farm 1. Challenge studies, VNT towards the relevant strains or testing of the cell-mediated immunity are needed to determine whether these animals are indeed naïve or have been primed [13]. However, none of these antibody negative sows revealed positive results in the RT-qPCR, indicating they should be of no concern.

### 4.3. Higher Response with Lower Value −2DPV

We tested the hypothesis that the increase in S/P value was correlated to the S/P value before vaccination in the sense that a high level of antibodies at the time of vaccination may inhibit the response to the booster vaccination. The test was based on the difference between the antibody levels before and two weeks after vaccination. The MFIA on both Farms 1 and 2 revealed a moderate negative monotonic correlation between the increase in antibody titre and the titre before vaccination. In contrast, the Idexx ELISA only revealed a very weak negative correlation on Farm 1 and no statistically significant relationship on Farm 2. Therefore, the change in S/P value after vaccination was not clearly related to the S/P value before vaccination. The reason for the differences between the results of the two tests are not clear. The antigen used in both tests was the Nucleoprotein (N), but this protein may have been processed differently during the manufacture of the two tests leading to differences in the recognition of different antibody isotypes and different epitopes exposed, etc.

### 4.4. Agreement between Idexx ELISA and MFIA

First, when comparing the two assays, the dynamic range and respective cut-off values must be considered in the assumption of any bias. This study detected a significant difference between the mean values before vaccination and between medians after vaccination on Farm 2 only. Overall, there was a very strong positive monotonic relationship between the quantitative results obtained by the Idexx ELISA and MFIA. The correlation seemed to be better prior to vaccination compared to two weeks after vaccination (*rs*: 0.89 vs. 0.83). This is the opposite of the finding in the study of Díaz et al. (2020), which found a better correlation after mass vaccination [13]. Therefore, the Spearman’s correlation analysis is, to some extent, considered unsuitable in the comparison of laboratory tests since it does not consider any systematic bias in the results. To eliminate the expected agreement purely by chance between the two assays, Cohen’s kappa value was calculated. The results indicated a substantial agreement between the two assays in all scenarios, ranging from κ = 0.65 two weeks after vaccination on Farm 1 to 0.77 before vaccination, also on Farm 1. The κ-values also indicated better agreement between the two serological assays before mass vaccination compared with two weeks after vaccination. This study revealed slightly higher κ-values than previously found by Díaz et al. (2020) [13]. However, the values might represent a conservative estimate since the prevalence of sows with antibodies towards PRRSV-1 in this study was high, making it more difficult to obtain a high κ -value. The presented κ -values should only be compared with other studies about PRRSV-vaccinated sows, otherwise, the κ-value may be misleading.

The results of this study revealed that to achieve the full advantage of PRRSV sow mass vaccination, i.e., to obtain PRRS virus-free nursery units, compliance towards the basic rules for effective PRRSV control is required. No negative side effects of the MLV PRRS vaccination were seen in these two herds, probably because the most vulnerable age groups were exempted from vaccination. It could be interesting to investigate whether a killed PRRS vaccine could initiate a similar immunological booster response as the MLV vaccine.

## Figures and Tables

**Table 1:** RT-qPCR. PRRS virus-positive samples in stables in the two nursery herds (N1 and N2) tested by RT-qPCR. In total, 236 pooled OF samples from nursery pigs before sow mass vaccination (−2DPV), two weeks after vaccination (WPV2) or 12 weeks after vaccination (WPV12) were tested.

Time	Herd	*n* of Stables/Approx. Pigs Tested	*n* of Stables with Positive Samples	
			PRRSV-1	PRRSV-2
−2DPV	N1	10/2000	1	2
	N2	10/2000	0	1
WPV2	N1	13/2600	0	0
	N2	8/1600	0	1
WPV12	N1	13/2600	0	4
	N2	5/1000	2	0

**Table 2:** Distribution of Seronegative Animals. Number of sows from Farms 1 (F1) and 2 (F2) that tested negative in the serological assays (Idexx ELISA and MFIA) before (−2DPV) and after (WPV2) mass vaccination with an MLV PRRSV-1 vaccine and by parity. Columns representing sample-to-positive ratios (S/P) for negative animals in both assays are included (*n* = total number of tested sows in each parity. “Both” refer to if Idexx ELISA and MFIA agrees with the serological PRRSV-1-status of the sow before and after mass vaccination. “0” represents PRRSV type indistinguishable values of MFIA. “+PRRSV-2” indicates sows with a ratio indicating dominance of PRRSV-2 antibodies. “Neg → Pos” indicates seroconverting animals, “Neg → Neg” indicates remaining negative antibodies, “Pos → Neg” indicates sows that shift from seropositive to seronegative and “IC” indicates inconclusive type of antibodies.

	F1							F2						
Parity	Sows (*n*)	Idexx ELISA		MFIA		Both		Sows (*n*)	Idexx ELISA		MFIA		Both	
		−2DPV	WPV2	−2DPV	WPV2	−2DPV	WPV2		−2DPV	WPV2	−2DPV	WPV2	−2DPV	WPV2
1	21	3	0	5	1	3	0	30	0	1	2	1	0	1
2	15	6	3	6	3	6	2	21	1	0	1	0	1	0
3	32	3	0	3	0	2	0	34	6	1	5	0	4	0
4	27	5	1	4	1	3	1	21	1	0	2	0	1	0
5	15	1	0	1	0	1	0	11	1	0	1	0	1	0
6	8	0	0	0	0	0	0	3	0	0	0	0	0	0
7	2	0	0	0	0	0	0	0	0	0	0	0	0	0
Total	120	18	4	19	5	15	3	120	9	2	11	1	7	1
0	-	-	-	7	10	-	-	-	-	-	4	11	-	-
+PRRSV-2	-	-	-	1	3	-	-	-	-	-	2	0	-	-
Neg → Pos	-	14		13		9		-	8		9		6	
Neg → Neg	-	4		4		3		-	1		1		0	
Pos → Neg	-	0		0		0		-	1		0		0	
IC → Pos	-	-		5		-		-	-		3		-	
IC → Neg	-	-		0		-		-	-		0		-	
Pos → IC	-	-		9		-		-	-		9		-	
Neg → IC	-	-		1		-		-	-		1		-	

**Table 3:** Serological Assay Results, F1 + F2—Before and After Vaccination. Dichotomous PRRSV-1 S/P antibody results in Idexx ELISA and MFIA before and after PRRSV-1 MLV mass vaccination in the study of two herds.

	Idexx ELISA Positive	Idexx ELISA Negative	Sum
MFIA-positive	394	7	401
MFIA-negative	10	26	36
Sum	404	33	437

**Table 4:** Agreements between Idexx ELISA and MFIA. Cohen’s kappa agreement coefficient (κ) between PRRSV-1 S/P antibody results in Idexx ELISA and ratio in MFIA before (−2DPV) and after PRRSV-1 MLV mass vaccination (WPV2) and in total for both serological assays in the study of two herds. Furthermore, standard error (*SE*), 95% confidence intervals (95% CI), observed agreements in numerical values and proportion and observed agreements by chance in numerical values and proportion.

	*κ*	*SE*	95% CI	Observed Agreements	Agreements by Chance
−2DPV	0.74	0.07	0.60–0.87	213 (94.25%)	176.2 (77.95%)
WPV2	0.66	0.16	0.34–0.97	207 (98.10%)	199.3 (94.47%)
Total	0.73	0.06	0.61–0.85	420 (96.11%)	373.4 (85.45%)

## Data Availability

The data presented in this study are available at this article and Table S3.

## Supplementary Materials

The following are available online at https://www.mdpi.com/article/10.3390/vaccines9101057/s1, Figure S1: PRRSV MLV vaccine doses sold in DK. Figure S2: Flow of pigs between the herds in the study. Table S1: RT-qPCR and serological assay results. Table S2: Dichotomous results of serological assays. Table S3: Spearman’s Correlation. Table S4: Cohen’s kappa agreement.
