# Supplementary material for: Impacts of Quarterly Sow Mass Vaccination with a Porcine Reproductive and Respiratory Syndrome Virus Type 1 (PRRSV-1) Modified Live Vaccine in Two Herds
Source: Vaccines (Basel). 2021 Sep 23;9(10):1057. doi: 10.3390/vaccines9101057 (PMC8537578; doi:10.3390/vaccines9101057)
Supplement: Supplementary file 1 [file vaccines-09-01057-s001.zip › vaccines-1321514-supplementary.pdf]

Figure S1 – PRRSV MLV vaccine doses sold in DK. Doses (y-axis, expressed x1000) of sold PRRSV MLV vaccines in Denmark from January 2017 until January 2021 (x-axis).

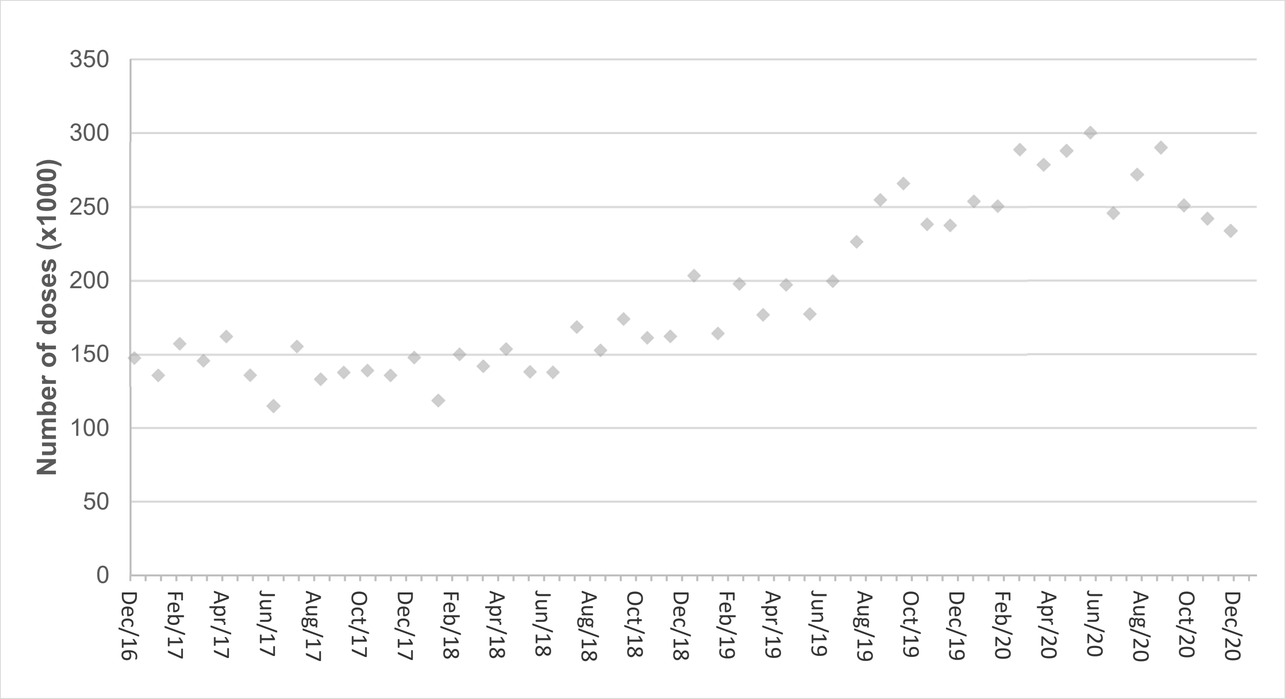

Figure S2 – Flow of pigs in between the herds in the study.

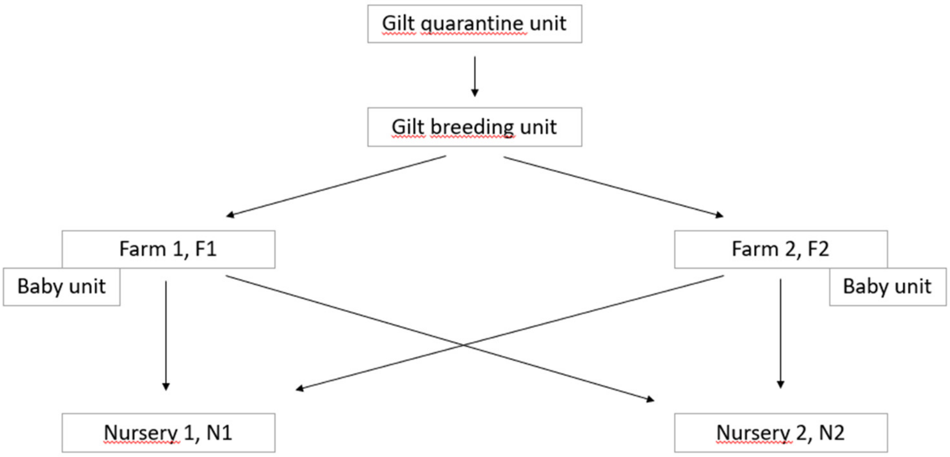

Table S1 – RT-qPCR and serologic assay (Idexx ELISA and MFIA) results. Herd are either farm 1 (F1) or 2 (F2), or nursery herd 1 (N1) or 2 (N2). Sampling date are given, and vaccination date for the sow herds with Porcilis PRRS was 05-12-2019. Number corresponds to either sow number in sow farms or section in nursery herds. Material are B = blood, Y= udder wipe, S = oral fluid. Remark corresponds to the detected PRRS virus.

Herd	Sampling date	Number	Material	Result, Cq	Remark	Idexx ELISA, S/P	MFIA-PRRSV1, S/P	MFIA-PRRSV2, S/P	MFIA-ratio
F1	03-12-2019	3602	B	0		0.76	0.49	0.25	-0.7
F1	03-12-2019	3989	B	0		0.84	0.2	0.3	0.4
F1	03-12-2019	3990	B	0		1.27	0.58	0.49	-0.2
F1	03-12-2019	3991	B	0		1.25	0.49	0.43	-0.1

F1	03-12-2019	3992	B	0	0.54	0.06	0.19	-
F1	03-12-2019	3993	B	0	1.26	0.62	0.58	-0.1
F1	03-12-2019	3994	B	0	1.13	0.58	0.52	-0.1
F1	03-12-2019	3995	B	0	1.8	0.95	0.85	-0.1
F1	03-12-2019	3996	B	0	0.73	0.37	0.23	-0.5
F1	03-12-2019	3997	B	0	1.32	0.61	0.51	-0.2
F1	03-12-2019	3999	B	0	0.94	0.39	0.39	0
F1	03-12-2019	4000	B	0	1.24	0.53	0.55	0
F1	03-12-2019	1984	B	0	0.48	0.32	0.22	-0.4
F1	03-12-2019	1478	B	0	2.15	1.15	0.99	-0.1
F1	03-12-2019	1885	B	0	1.57	0.84	0.6	-0.3
F1	03-12-2019	1207	B	0	1.54	0.78	0.64	-0.2
F1	03-12-2019	2076	B	0	1.1	0.94	0.69	-0.3
F1	03-12-2019	2050	B	0	1.64	1.02	0.73	-0.3
F1	03-12-2019	1996	B	0	1.21	0.78	0.61	-0.2
F1	03-12-2019	1566	B	0	1.1	0.79	0.63	-0.2
F1	03-12-2019	10196	B	0	1.2	0.76	0.59	-0.3
F1	03-12-2019	2011	B	0	1.49	0.93	0.84	-0.1
F1	03-12-2019	2504	B	0	1.83	0.95	0.82	-0.1
F1	03-12-2019	2484	B	0	0.9	0.63	0.52	-0.2
F1	03-12-2019	2441	B	0	0.99	0.64	0.39	-0.5
F1	03-12-2019	2461	B	0	2.02	1.06	0.86	-0.2
F1	03-12-2019	3006	B	0	0.98	0.6	0.37	-0.5
F1	03-12-2019	3059	B	0	1.72	0.9	0.88	0
F1	03-12-2019	3087	B	0	1.71	1.01	0.73	-0.3
F1	03-12-2019	3123	B	0	0.94	0.46	0.34	-0.3
F1	03-12-2019	3117	B	0	1.11	1.04	0.93	-0.1
F1	03-12-2019	3020	B	0	0.54	0.23	0.12	-
F1	03-12-2019	3572	B	0	0.72	0.48	0.23	-0.7
F1	03-12-2019	3099	B	0	1.23	0.95	0.65	-0.4
F1	03-12-2019	3049	B	0	1.43	0.82	0.65	-0.2
F1	03-12-2019	2950	B	0	1.16	0.75	0.43	-0.6
F1	03-12-2019	3558	B	0	1.73	0.77	0.43	-0.6
F1	03-12-2019	2492	B	0	1.77	1.21	0.99	-0.2
F1	03-12-2019	1970	B	0	0.26	0.21	0.02	-
F1	03-12-2019	2576	B	0	1.43	0.72	0.52	-0.3
F1	03-12-2019	3617	B	0	1.45	0.76	0.67	-0.1
F1	03-12-2019	3016	B	0	0.81	0.81	0.74	-0.1
F1	03-12-2019	3032	B	0	1.51	1	0.76	-0.3
F1	03-12-2019	3131	B	0	1.26	0.52	0.35	-0.4
F1	03-12-2019	2564	B	0	0.81	0.51	0.3	-0.5
F1	03-12-2019	3142	B	0	0.75	0.62	0.19	-1.2
F1	03-12-2019	3544	B	0	0.29	0.11	0.11	-
F1	03-12-2019	3056	B	0	0.99	0.55	0.53	0
F1	03-12-2019	2539	B	0	1.2	0.91	0.41	-0.8
F1	03-12-2019	3608	B	0	0.09	0.07	0.04	-
F1	03-12-2019	3446	B	0	1.49	1.11	0.79	-0.3
F1	03-12-2019	2530	B	0	0.64	0.52	0.28	-0.6
F1	03-12-2019	2354	B	0	1.07	0.86	0.44	-0.7
F1	03-12-2019	3560	B	0	0.24	0.16	0.08	-
F1	03-12-2019	2480	B	0	1.69	1.07	0.85	-0.2

F1	03-12-2019	3057	B	0	2.01	1.17	0.94	-0.2
F1	03-12-2019	2542	B	0	1.4	0.99	0.56	-0.6
F1	03-12-2019	3063	B	0	1.74	1.12	0.59	-0.6
F1	03-12-2019	3046	B	0	0.3	0.33	0.14	-0.8
F1	03-12-2019	3581	B	0	0.29	0.21	0.15	-
F1	03-12-2019	3086	B	0	0.69	0.32	0.19	-0.5
F1	03-12-2019	2459	B	0	1.47	0.97	0.78	-0.2
F1	03-12-2019	3546	B	0	0.16	0.03	0.04	-
F1	03-12-2019	3019	B	0	0.92	0.64	0.35	-0.6
F1	03-12-2019	1943	B	0	0.68	0.69	0.41	-0.5
F1	03-12-2019	1975	B	0	1	0.68	0.35	-0.7
F1	03-12-2019	3961	B	0	1.37	0.8	0.59	-0.3
F1	03-12-2019	3025	B	0	1.63	0.93	0.54	-0.5
F1	03-12-2019	2485	B	0	0.29	0.33	0.15	-0.8
F1	03-12-2019	1957	B	0	0.81	0.6	0.51	-0.2
F1	03-12-2019	2440	B	0	2.29	0.91	0.95	0
F1	03-12-2019	2051	B	0	1.52	0.95	0.71	-0.3
F1	03-12-2019	3074	B	0	0.9	0.59	0.25	-0.9
F1	03-12-2019	2960	B	0	0.34	0.04	0.15	-
F1	03-12-2019	3910	B	0	1.48	0.99	0.7	-0.3
F1	03-12-2019	2434	B	0	0.69	0.54	0.18	-1.1
F1	03-12-2019	1324	B	0	1.48	0.85	0.56	-0.4
F1	03-12-2019	2972	B	0	1.85	1.09	0.82	-0.3
F1	03-12-2019	1500	B	0	0.97	0.5	0.34	-0.4
F1	03-12-2019	2980	B	0	1.61	1.04	0.77	-0.3
F1	03-12-2019	1959	B	0	1.8	1.15	0.7	-0.5
F1	03-12-2019	2490	B	0	0.3	0.18	0.13	-
F1	03-12-2019	2489	B	0	1.97	1.18	0.65	-0.6
F1	03-12-2019	3958	B	0	0.39	0.19	0.07	-
F1	03-12-2019	2436	B	0	0.89	0.63	0.34	-0.6
F1	03-12-2019	2986	B	0	1.65	1.06	0.72	-0.4
F1	03-12-2019	3101	B	0	1.78	1.15	0.95	-0.2
F1	03-12-2019	2472	B	0	0.35	0.15	0.1	-
F1	03-12-2019	2007	B	0	1.96	1.24	1.15	-0.1
F1	03-12-2019	1954	B	0	1.5	0.93	0.65	-0.4
F1	03-12-2019	3964	B	0	1.51	1.16	0.95	-0.2
F1	03-12-2019	1484	B	0	1.03	0.7	0.35	-0.7
F1	03-12-2019	2449	B	0	0.5	0.21	0.14	-
F1	03-12-2019	2349	B	0	0.23	0.16	0.08	-
F1	03-12-2019	1475	B	0	0.92	0.56	0.41	-0.3
F1	03-12-2019	3072	B	0	0.71	0.64	0.34	-0.6
F1	03-12-2019	3955	B	0	0.99	0.51	0.45	-0.1
F1	03-12-2019	2426	B	0	0.38	0.28	0.1	-1.1
F1	03-12-2019	3957	B	0	1.13	0.79	0.53	-0.4
F1	03-12-2019	3953	B	0	2.35	1.5	1.46	0
F1	03-12-2019	3023	B	0	0.32	0.14	0.09	-
F1	03-12-2019	2432	B	0	0.5	0.59	0.23	-0.9
F1	03-12-2019	2355	B	0	2.31	1.23	1.17	0
F1	03-12-2019	2466	B	0	1.04	0.97	0.37	-1
F1	03-12-2019	3956	B	0	0.17	0.03	0.06	-
F1	03-12-2019	2381	B	0	1.21	0.75	0.54	-0.3

F1	03-12-2019	3547	B	0	1.05	0.46	0.33	-0.3
F1	03-12-2019	3551	B	0	0.31	0.22	0.17	-
F1	03-12-2019	3013	B	0	1.34	0.89	0.49	-0.6
F1	03-12-2019	1151	B	0	1.5	1.08	0.61	-0.6
F1	03-12-2019	2973	B	0	0.91	0.44	0.32	-0.3
F1	03-12-2019	3549	B	0	0.45	0.27	0.18	-0.4
F1	03-12-2019	3954	B	0	0.21	0.17	0.09	-
F1	03-12-2019	3034	B	0	1.24	1.16	0.82	-0.3
F1	03-12-2019	1438	B	0	0.98	0.76	0.58	-0.3
F1	03-12-2019	3553	B	0	1	0.54	0.39	-0.3
F1	03-12-2019	1966	B	0	1.29	0.87	0.65	-0.3
F1	03-12-2019	2997	B	0	1.84	1.19	1.04	-0.1
F1	03-12-2019	3550	B	0	1.26	0.92	0.64	-0.4
F1	03-12-2019	3959	B	0	0.47	0.22	0.13	-
F2	03-12-2019	9013	B	0	0.89	0.52	0.38	-0.3
F2	03-12-2019	9011	B	0	1.02	0.56	0.49	-0.1
F2	03-12-2019	9012	B	0	1.43	0.98	0.88	-0.1
F2	03-12-2019	9020	B	0	0.4	0.17	0.15	-
F2	03-12-2019	9018	B	0	1.25	0.85	0.58	-0.4
F2	03-12-2019	9015	B	0	1.31	0.93	0.7	-0.3
F2	03-12-2019	9009	B	0	0.75	0.3	0.2	-0.4
F2	03-12-2019	9017	B	0	1.36	0.75	0.57	-0.3
F2	03-12-2019	9010	B	0	0.88	0.38	0.31	-0.2
F2	03-12-2019	9019	B	0	0.85	0.46	0.32	-0.4
F2	03-12-2019	9016	B	0	0.75	0.42	0.35	-0.2
F2	03-12-2019	9014	B	0	1.23	0.68	0.56	-0.2
F2	03-12-2019	6964	B	0	0.88	0.57	0.4	-0.4
F2	03-12-2019	6532	B	0	0.57	0.42	0.29	-0.4
F2	03-12-2019	6902	B	0	0.71	0.43	0.2	-0.8
F2	03-12-2019	7521	B	0	1.41	0.76	0.47	-0.5
F2	03-12-2019	7485	B	0	0.89	0.47	0.35	-0.3
F2	03-12-2019	7880	B	0	1.08	0.46	0.22	-0.7
F2	03-12-2019	7453	B	0	1.71	0.95	0.77	-0.2
F2	03-12-2019	7018	B	0	1.1	0.72	0.47	-0.4
F2	03-12-2019	7399	B	0	0.55	0.33	0.17	-0.7
F2	03-12-2019	7911	B	0	0.34	0.16	0.07	-
F2	03-12-2019	7901	B	0	0.49	0.25	0.17	-0.4
F2	03-12-2019	7886	B	0	0.35	0.34	0.18	-0.6
F2	03-12-2019	7423	B	0	1.56	1.01	0.6	-0.5
F2	03-12-2019	7425	B	0	1.42	0.96	0.58	-0.5
F2	03-12-2019	7732	B	0	0.14	0.04	0.01	-
F2	03-12-2019	6533	B	0	0.86	0.29	0.31	0.1
F2	03-12-2019	7456	B	0	0.42	0.19	0.12	-
F2	03-12-2019	7885	B	0	1.69	0.95	0.81	-0.2
F2	03-12-2019	8485	B	0	1.49	0.8	0.61	-0.3
F2	03-12-2019	7424	B	0	2.05	1.26	0.88	-0.4
F2	03-12-2019	7361	B	0	1.3	0.9	0.82	-0.1
F2	03-12-2019	7844	B	0	0.62	0.35	0.23	-0.4
F2	03-12-2019	6945	B	0	0.37	0.19	0.11	-
F2	03-12-2019	7920	B	0	1.2	0.92	0.62	-0.4
F2	03-12-2019	7883	B	0	1.97	1.47	1.35	-0.1

F2	03-12-2019	6257	B	0	0.98	0.64	0.5	-0.2
F2	03-12-2019	6962	B	0	0.91	0.51	0.2	-0.9
F2	03-12-2019	7388	B	0	1.68	1.2	0.97	-0.2
F2	03-12-2019	6983	B	0	1.5	0.39	0.25	-0.4
F2	03-12-2019	6931	B	0	0.55	0.32	0.12	-1
F2	03-12-2019	7503	B	0	0.37	0.16	0.11	-
F2	03-12-2019	7887	B	0	0.69	0.15	0.09	-
F2	03-12-2019	6924	B	0	1.06	0.74	0.5	-0.4
F2	03-12-2019	6892	B	0	1.97	1.21	0.96	-0.2
F2	03-12-2019	7513	B	0	1.86	1.26	1.14	-0.1
F2	03-12-2019	7409	B	0	1.57	1.09	0.82	-0.3
F2	03-12-2019	7982	B	0	0.89	0.34	0.23	-0.4
F2	03-12-2019	7413	B	0	0.82	0.6	0.28	-0.7
F2	03-12-2019	7909	B	0	1.04	0.54	0.3	-0.6
F2	03-12-2019	7450	B	0	1.32	0.83	0.67	-0.2
F2	03-12-2019	8501	B	0	1.62	1.07	0.93	-0.1
F2	03-12-2019	8516	B	0	0.93	0.69	0.42	-0.5
F2	03-12-2019	7861	B	0	1.49	0.84	0.5	-0.5
F2	03-12-2019	8467	B	0	0.25	0.07	0.11	-
F2	03-12-2019	7951	B	0	1.21	0.34	0.14	-0.9
F2	03-12-2019	8490	B	0	1.79	0.84	0.54	-0.4
F2	03-12-2019	8498	B	0	1.2	0.43	0.29	-0.4
F2	03-12-2019	8574	B	0	1.06	0.69	0.17	-1.4
F2	03-12-2019	7831	B	0	1	0.47	0.4	-0.2
F2	03-12-2019	7858	B	0	1.24	0.8	0.37	-0.8
F2	03-12-2019	8513	B	0	0.55	0.28	0.22	-0.3
F2	03-12-2019	8439	B	0	1.2	0.7	0.4	-0.6
F2	03-12-2019	7912	B	0	1.47	1.09	0.79	-0.3
F2	03-12-2019	7819	B	0	1.04	0.47	0.38	-0.2
F2	03-12-2019	8434	B	0	0.97	0.36	0.32	-0.1
F2	03-12-2019	8483	B	0	1.17	0.7	0.38	-0.6
F2	03-12-2019	8423	B	0	0.53	0.26	0.07	-1.3
F2	03-12-2019	8507	B	0	1.45	0.87	0.6	-0.4
F2	03-12-2019	8430	B	0	1.42	0.71	0.48	-0.4
F2	03-12-2019	6916	B	0	1.86	1.12	1.07	0
F2	03-12-2019	7868	B	0	1.79	0.86	0.15	-1.8
F2	03-12-2019	7888	B	0	1.31	0.68	0.56	-0.2
F2	03-12-2019	8450	B	0	1.23	0.59	0.53	-0.1
F2	03-12-2019	7846	B	0	1.58	1.17	1.03	-0.1
F2	03-12-2019	7345	B	0	1.09	0.6	0.28	-0.8
F2	03-12-2019	7408	B	0	0.96	0.37	0.26	-0.3
F2	03-12-2019	7877	B	0	1.55	0.8	0.55	-0.4
F2	03-12-2019	7894	B	0	0.42	0.66	0.21	-1.1
F2	03-12-2019	7904	B	0	0.38	0.33	0.15	-0.8
F2	03-12-2019	7827	B	0	0.21	0.09	0.04	-
F2	03-12-2019	7931	B	0	2.4	1.37	1.24	-0.1
F2	03-12-2019	6918	B	0	1.33	1.03	0.71	-0.4
F2	03-12-2019	7836	B	0	1.48	1	0.75	-0.3
F2	03-12-2019	8503	B	0	0.92	0.47	0.29	-0.5
F2	03-12-2019	7825	B	0	1.54	0.82	0.61	-0.3
F2	03-12-2019	8461	B	0	0.57	0.49	0.3	-0.5

F2	03-12-2019	7443	B	0	1.11	0.87	0.49	-0.6
F2	03-12-2019	7404	B	0	0.6	0.48	0.28	-0.5
F2	03-12-2019	8419	B	0	0.63	0.33	0.15	-0.8
F2	03-12-2019	7873	B	0	0.86	0.48	0.41	-0.1
F2	03-12-2019	7403	B	0	0.77	0.33	0.08	-1.4
F2	03-12-2019	7853	B	0	1.99	1.21	0.8	-0.4
F2	03-12-2019	7770	B	0	1.45	0.89	0.6	-0.4
F2	03-12-2019	7740	B	0	0.05	0.07	0.07	-
F2	03-12-2019	8412	B	0	0.79	0.26	0.19	-0.3
F2	03-12-2019	7899	B	0	0.79	0.62	0.4	-0.4
F2	03-12-2019	7856	B	0	1.24	0.87	0.59	-0.4
F2	03-12-2019	7448	B	0	0.6	0.48	0.2	-0.9
F2	03-12-2019	8489	B	0	0.74	0.45	0.31	-0.4
F2	03-12-2019	8592	B	0	1.14	0.82	0.31	-1
F2	03-12-2019	8982	B	0	1.17	0.67	0.6	-0.1
F2	03-12-2019	8981	B	0	1.35	0.64	0.49	-0.3
F2	03-12-2019	8978	B	0	1.76	1.01	0.97	0
F2	03-12-2019	8983	B	0	1.75	1.01	0.86	-0.2
F2	03-12-2019	8979	B	0	0.57	0.32	0.22	-0.4
F2	03-12-2019	8977	B	0	1.98	1.28	1.27	0
F2	03-12-2019	8973	B	0	1.47	0.69	0.59	-0.1
F2	03-12-2019	8968	B	0	0.92	0.7	0.59	-0.2
F2	03-12-2019	8970	B	0	1.2	0.81	0.5	-0.5
F2	03-12-2019	8975	B	0	1.2	0.49	0.47	0
F2	03-12-2019	8976	B	0	2.05	1.25	1.15	-0.1
F2	03-12-2019	8971	B	0	0.78	0.51	0.23	-0.8
F2	03-12-2019	8974	B	0	0.44	0.27	0.23	-0.2
F2	03-12-2019	8966	B	0	1.18	0.71	0.53	-0.3
F2	03-12-2019	8967	B	0	1.21	0.64	0.45	-0.4
F2	03-12-2019	8969	B	0	0.44	0.07	0.19	-
F2	03-12-2019	8972	B	0	1.57	0.77	0.6	-0.2
F2	03-12-2019	8921	B	0	0.99	0.36	0.45	0.2
F1	19-12-2019	3602	B	0	1.44	0.99	0.65	-0.4
F1	19-12-2019	3989	B	0	0.75	0.18	0.22	-
F1	19-12-2019	3990	B	0	1.97	1.04	0.93	-0.1
F1	19-12-2019	3991	B	0	1.39	0.67	0.57	-0.2
F1	19-12-2019	3992	B	0	1.95	0.81	0.99	0.2
F1	19-12-2019	3993	B	0	1.51	0.86	0.76	-0.1
F1	19-12-2019	3994	B	0	1.52	0.87	0.75	-0.1
F1	19-12-2019	3995	B	0	1.93	1	0.88	-0.1
F1	19-12-2019	3996	B	0	1.36	0.88	0.59	-0.4
F1	19-12-2019	3997	B	0	1.43	0.82	0.71	-0.1
F1	19-12-2019	3999	B	0	1.84	0.84	1.09	0.3
F1	19-12-2019	4000	B	0	1.18	0.79	0.67	-0.2
F1	19-12-2019	1984	B	0	0.76	0.64	0.41	-0.5
F1	19-12-2019	1478	B	0	2.21	1.31	1.12	-0.2
F1	19-12-2019	1885	B	0	2.53	1.25	1.14	-0.1
F1	19-12-2019	1207	B	0	2.07	1.1	1	-0.1
F1	19-12-2019	2076	B	0	1.02	0.9	0.6	-0.4
F1	19-12-2019	2050	B	0	2.1	1.24	1.07	-0.1
F1	19-12-2019	1996	B	0	0.86	0.77	0.58	-0.3

F1	19-12-2019	1566	B	0	1.55	0.89	0.81	-0.1
F1	19-12-2019	10196	B	0	1.91	1.1	1.04	0
F1	19-12-2019	2011	B	0	1.81	1.19	1.09	-0.1
F1	19-12-2019	2504	B	0	2.26	1.19	1.02	-0.1
F1	19-12-2019	2484	B	0	0.82	0.67	0.54	-0.2
F1	19-12-2019	2441	B	0	0.98	0.67	0.43	-0.4
F1	19-12-2019	2461	B	0	2.73	1.16	1.18	0
F1	19-12-2019	3006	B	0	1.58	1.21	0.96	-0.2
F1	19-12-2019	3059	B	0	1.73	1.04	0.92	-0.1
F1	19-12-2019	3087	B	0	1.69	1.13	0.73	-0.4
F1	19-12-2019	3123	B	0	0.78	0.47	0.32	-0.4
F1	19-12-2019	3117	B	0	2.09	1.16	1.1	-0.1
F1	19-12-2019	3020	B	0	2.08	1.19	0.94	-0.2
F1	19-12-2019	3572	B	0	1.21	1	0.54	-0.6
F1	19-12-2019	3099	B	0	2.66	1.14	1.15	0
F1	19-12-2019	3049	B	0	0.87	0.74	0.58	-0.2
F1	19-12-2019	2950	B	0	2.1	0.93	0.89	0
F1	19-12-2019	3558	B	0	2.1	1.21	0.77	-0.5
F1	19-12-2019	2492	B	0	1.54	1.05	0.8	-0.3
F1	19-12-2019	1970	B	0	0.42	0.35	0.02	-2.8
F1	19-12-2019	2576	B	0	1.74	1.01	0.83	-0.2
F1	19-12-2019	3617	B	0	1.47	0.94	0.82	-0.1
F1	19-12-2019	3016	B	0	1.41	1.09	0.9	-0.2
F1	19-12-2019	3032	B	0	2.1	1.18	1.18	0
F1	19-12-2019	3131	B	0	1.97	1.12	0.99	-0.1
F1	19-12-2019	2564	B	0	0.66	0.58	0.32	-0.6
F1	19-12-2019	3142	B	0	1.3	0.96	0.53	-0.6
F1	19-12-2019	3544	B	0	0.25	0.14	0.11	-
F1	19-12-2019	3056	B	0	1.92	1.13	1.07	-0.1
F1	19-12-2019	2539	B	0	2.48	1.26	1.2	-0.1
F1	19-12-2019	3608	B	0	0.33	0.38	0.23	-0.5
F1	19-12-2019	3446	B	0	1.67	1.17	1.01	-0.1
F1	19-12-2019	2530	B	0	1.71	1.32	1.14	-0.1
F1	19-12-2019	2354	B	0	1.25	1.05	0.65	-0.5
F1	19-12-2019	3560	B	0	0.22	0.18	0.09	-
F1	19-12-2019	2480	B	0	1.97	1.25	1.11	-0.1
F1	19-12-2019	3057	B	0	2.51	1.17	1.17	0
F1	19-12-2019	2542	B	0	1.32	1.06	0.69	-0.4
F1	19-12-2019	3063	B	0	2.45	1.19	1.24	0
F1	19-12-2019	3046	B	0	0.88	0.88	0.5	-0.6
F1	19-12-2019	3581	B	0	1.17	0.96	0.74	-0.3
F1	19-12-2019	3086	B	0	2.17	1.14	1.06	-0.1
F1	19-12-2019	2459	B	0	1.76	1.12	1.05	-0.1
F1	19-12-2019	3546	B	0	0.44	0.15	0.14	-
F1	19-12-2019	3019	B	0	1.6	0	0	NA
F1	19-12-2019	1943	B	0	0.82	1.04	0.74	-0.3
F1	19-12-2019	1975	B	0	1.5	0.92	0.49	-0.6
F1	19-12-2019	3961	B	0	1.66	1.05	0.91	-0.1
F1	19-12-2019	3025	B	0	1.55	0.85	0.61	-0.3
F1	19-12-2019	2485	B	0	0.74	0.72	0.36	-0.7
F1	19-12-2019	1957	B	0	0.92	0.9	0.83	-0.1

F1	19-12-2019	2440	B	0	2.44	0.78	0.91	0.1
F1	19-12-2019	2051	B	0	1.85	1.18	1.01	-0.2
F1	19-12-2019	3074	B	0	1	0.78	0.4	-0.7
F1	19-12-2019	2960	B	0	2.32	1.11	1.1	0
F1	19-12-2019	3910	B	0	1.42	1.18	0.9	-0.3
F1	19-12-2019	2434	B	0	1.5	0.91	0.43	-0.8
F1	19-12-2019	1324	B	0	1.4	1.04	0.73	-0.4
F1	19-12-2019	2972	B	0	2.18	0.54	0.5	-0.1
F1	19-12-2019	1500	B	0	1.21	0.81	0.62	-0.3
F1	19-12-2019	2980	B	0	2.2	1.25	1.14	-0.1
F1	19-12-2019	1959	B	0	2.44	1.26	1.02	-0.2
F1	19-12-2019	2490	B	0	0.63	0.58	0.31	-0.6
F1	19-12-2019	2489	B	0	2.05	1.13	0.79	-0.4
F1	19-12-2019	3958	B	0	0.71	0.44	0.15	-1.1
F1	19-12-2019	2436	B	0	1.64	1.09	0.85	-0.2
F1	19-12-2019	2986	B	0	1.86	1.06	0.8	-0.3
F1	19-12-2019	3101	B	0	2.06	0.92	0.86	-0.1
F1	19-12-2019	2472	B	0	1.19	0.59	0.37	-0.5
F1	19-12-2019	2007	B	0	2.76	1.26	1.3	0
F1	19-12-2019	1954	B	0	2.16	0	0	NA
F1	19-12-2019	3964	B	0	1.85	1.04	1.04	0
F1	19-12-2019	1484	B	0	1.44	0.99	0.62	-0.5
F1	19-12-2019	2449	B	0	1.63	0.85	0.65	-0.3
F1	19-12-2019	2349	B	0	0.27	0.19	0.1	-
F1	19-12-2019	1475	B	0	1.03	0.66	0.49	-0.3
F1	19-12-2019	3072	B	0	1.13	0.74	0.41	-0.6
F1	19-12-2019	3955	B	0	1.58	0.83	0.57	-0.4
F1	19-12-2019	2426	B	0	1.1	0.73	0.31	-0.9
F1	19-12-2019	3957	B	0	1.34	0.91	0.62	-0.4
F1	19-12-2019	3953	B	0	2.52	1.43	1.35	-0.1
F1	19-12-2019	3023	B	0	0.56	0.49	0.3	-0.5
F1	19-12-2019	2432	B	0	0.71	0.73	0.35	-0.7
F1	19-12-2019	2355	B	0	2.68	1.29	1.22	-0.1
F1	19-12-2019	2466	B	0	1	0	0	NA
F1	19-12-2019	3956	B	0	1.74	1.28	1.02	-0.2
F1	19-12-2019	2381	B	0	1.92	0	0	NA
F1	19-12-2019	3547	B	0	0.97	0.63	0.39	-0.5
F1	19-12-2019	3551	B	0	1.24	0.95	0.45	-0.7
F1	19-12-2019	3013	B	0	1.92	1.27	0.92	-0.3
F1	19-12-2019	1151	B	0	2.1	1.26	0.96	-0.3
F1	19-12-2019	2973	B	0	1.09	0.56	0.36	-0.4
F1	19-12-2019	3549	B	0	1	0	0	NA
F1	19-12-2019	3954	B	0	2.22	1.13	1.01	-0.1
F1	19-12-2019	3034	B	0	2.12	1.29	1.14	-0.1
F1	19-12-2019	1438	B	0	1.44	1.11	0.92	-0.2
F1	19-12-2019	3553	B	0	1.01	0.75	0.48	-0.4
F1	19-12-2019	1966	B	0	1.89	1.08	0.92	-0.2
F1	19-12-2019	2997	B	0	2.32	1.29	1.18	-0.1
F1	19-12-2019	3550	B	0	1.59	1.12	0.9	-0.2
F1	19-12-2019	3959	B	0	2.06	1.29	1.07	-0.2
F2	19-12-2019	9013	B	0	1.57	0.99	0.72	-0.3

F2	19-12-2019	9011	B	0	1.99	1.18	0.83	-0.3
F2	19-12-2019	9012	B	0	2.66	1.21	1.15	-0.1
F2	19-12-2019	9020	B	0	0.31	0.13	0.09	-
F2	19-12-2019	9018	B	0	1.49	1.08	0.9	-0.2
F2	19-12-2019	9015	B	0	1.28	0.83	0.58	-0.4
F2	19-12-2019	9009	B	0	1.86	1.09	0.77	-0.4
F2	19-12-2019	9017	B	0	1.31	1.01	0.74	-0.3
F2	19-12-2019	9010	B	0	1.1	0.49	0.35	-0.4
F2	19-12-2019	9019	B	0	1.33	0.74	0.5	-0.4
F2	19-12-2019	9016	B	0	0.67	0.37	0.25	-0.4
F2	19-12-2019	9014	B	0	1.33	0.79	0.59	-0.3
F2	19-12-2019	6964	B	0	2.72	0.99	0.93	-0.1
F2	19-12-2019	6532	B	0	0.53	0.39	0.25	-0.4
F2	19-12-2019	6902	B	0	1.8	0.85	0.6	-0.4
F2	19-12-2019	7521	B	0	2.58	1.25	1.15	-0.1
F2	19-12-2019	7485	B	0	0.8	0.39	0.27	-0.4
F2	19-12-2019	7880	B	0	1.96	1.25	0.99	-0.2
F2	19-12-2019	7453	B	0	2.73	1.14	1.12	0
F2	19-12-2019	7018	B	0	2.35	1.17	1.07	-0.1
F2	19-12-2019	7399	B	0	0.7	0.46	0.23	-0.7
F2	19-12-2019	7911	B	0	1.3	0.79	0.48	-0.5
F2	19-12-2019	7901	B	0	0.64	0.39	0.27	-0.4
F2	19-12-2019	7886	B	0	1.28	0.94	0.69	-0.3
F2	19-12-2019	7423	B	0	1.75	1.12	0.63	-0.6
F2	19-12-2019	7425	B	0	1.88	1.14	0.67	-0.5
F2	19-12-2019	7732	B	0	0.75	0.41	0.16	-0.9
F2	19-12-2019	6533	B	0	1.54	0.8	0.63	-0.2
F2	19-12-2019	7456	B	0	1.13	0.7	0.5	-0.3
F2	19-12-2019	7885	B	0	2.56	1.03	0.99	0
F2	19-12-2019	8485	B	0	2.94	1.27	1.23	0
F2	19-12-2019	7424	B	0	2.61	1.21	1.15	-0.1
F2	19-12-2019	7361	B	0	2.47	1.32	1.28	0
F2	19-12-2019	7844	B	0	2.08	1.3	1.1	-0.2
F2	19-12-2019	6945	B	0	0.94	0.7	0.43	-0.5
F2	19-12-2019	7920	B	0	1.52	0.84	0.5	-0.5
F2	19-12-2019	7883	B	0	2.61	1.52	1.44	-0.1
F2	19-12-2019	6257	B	0	1.53	1.06	0.86	-0.2
F2	19-12-2019	6962	B	0	2.24	1.14	0.98	-0.1
F2	19-12-2019	7388	B	0	2.42	1.21	1.16	0
F2	19-12-2019	6983	B	0	2.3	1.12	0.83	-0.3
F2	19-12-2019	6931	B	0	1.49	1.01	0.58	-0.6
F2	19-12-2019	7503	B	0	0.64	0.4	0.26	-0.4
F2	19-12-2019	7887	B	0	2.3	1.11	0.9	-0.2
F2	19-12-2019	6924	B	0	2.08	1.16	0.89	-0.3
F2	19-12-2019	6892	B	0	2.51	1.22	1.18	0
F2	19-12-2019	7513	B	0	2.53	1.34	1.29	0
F2	19-12-2019	7409	B	0	0.93	0.63	0.34	-0.6
F2	19-12-2019	7982	B	0	2.23	1.09	0.83	-0.3
F2	19-12-2019	7413	B	0	1.15	0.78	0.42	-0.6
F2	19-12-2019	7909	B	0	2.38	1.29	0.94	-0.3
F2	19-12-2019	7450	B	0	2.39	1.29	1.17	-0.1

F2	19-12-2019	8501	B	0	1.61	1.07	0.85	-0.2
F2	19-12-2019	8516	B	0	1.12	0.63	0.43	-0.4
F2	19-12-2019	7861	B	0	2.7	1.39	1.32	-0.1
F2	19-12-2019	8467	B	0	0.56	0.5	0.26	-0.6
F2	19-12-2019	7951	B	0	1.72	0.8	0.38	-0.7
F2	19-12-2019	8490	B	0	2.3	1.32	1.09	-0.2
F2	19-12-2019	8498	B	0	2	1.17	0.93	-0.2
F2	19-12-2019	8574	B	0	2.26	1.37	0.76	-0.6
F2	19-12-2019	7831	B	0	1.7	0.96	0.77	-0.2
F2	19-12-2019	7858	B	0	1.72	1.03	0.54	-0.7
F2	19-12-2019	8513	B	0	1.89	1.17	1.01	-0.2
F2	19-12-2019	8439	B	0	1.45	1.06	0.66	-0.5
F2	19-12-2019	7912	B	0	2.24	1.39	1.23	-0.1
F2	19-12-2019	7819	B	0	1.55	1.08	0.87	-0.2
F2	19-12-2019	8434	B	0	1.8	0.95	0.82	-0.1
F2	19-12-2019	8483	B	0	1.47	0.99	0.55	-0.6
F2	19-12-2019	8423	B	0	1.55	1.02	0.52	-0.7
F2	19-12-2019	8507	B	0	2.05	1.1	0.93	-0.21
F2	19-12-2019	8430	B	0	2.07	1.07	0.93	-0.1
F2	19-12-2019	6916	B	0	2.31	1.02	1.03	0
F2	19-12-2019	7868	B	0	2.1	1.2	0.52	-0.8
F2	19-12-2019	7888	B	0	2	1.01	0.95	-0.1
F2	19-12-2019	8450	B	0	2.23	1.03	1.02	0
F2	19-12-2019	7846	B	0	2.18	1.19	0.93	-0.2
F2	19-12-2019	7345	B	0	1.19	0.91	0.41	-0.8
F2	19-12-2019	7408	B	0	1.51	1.06	0.78	-0.3
F2	19-12-2019	7877	B	0	2.14	1.2	1.08	-0.1
F2	19-12-2019	7894	B	0	0.55	0.57	0.23	-0.9
F2	19-12-2019	7904	B	0	2.44	1.19	1.08	-0.1
F2	19-12-2019	7827	B	0	0.68	0.44	0.24	-0.6
F2	19-12-2019	7931	B	0	2.69	1.35	1.25	-0.1
F2	19-12-2019	6918	B	0	1.58	1.18	0.88	-0.3
F2	19-12-2019	7836	B	0	2.05	1.14	1.05	-0.1
F2	19-12-2019	8503	B	0	2.48	0.98	0.88	-0.1
F2	19-12-2019	7825	B	0	2.51	1.14	1.1	0
F2	19-12-2019	8461	B	0	1.85	1.06	0.9	-0.2
F2	19-12-2019	7443	B	0	1.39	0.96	0.58	-0.5
F2	19-12-2019	7404	B	0	0.82	0.55	0.33	-0.5
F2	19-12-2019	8419	B	0	1.67	1.18	0.67	-0.6
F2	19-12-2019	7873	B	0	1.9	1.02	0.69	-0.4
F2	19-12-2019	7403	B	0	1.63	1.03	0.28	-1.3
F2	19-12-2019	7853	B	0	2.74	1.4	1.3	-0.1
F2	19-12-2019	7770	B	0	1.54	0.93	0.63	-0.4
F2	19-12-2019	7740	B	0	0.21	0.32	0.15	-0.7
F2	19-12-2019	8412	B	0	1.95	1.1	0.91	-0.2
F2	19-12-2019	7899	B	0	1.82	1.24	1	-0.2
F2	19-12-2019	7856	B	0	1.93	1.02	0.85	-0.2
F2	19-12-2019	7448	B	0	1.47	0.96	0.53	-0.6
F2	19-12-2019	8489	B	0	0.92	0.56	0.35	-0.5
F2	19-12-2019	8592	B	0	1.42	0.84	0.39	-0.8
F2	19-12-2019	8982	B	0	1.84	1.08	0.93	-0.2

F2	19-12-2019	8981	B	0	1.91	0.83	0.63	-0.3
F2	19-12-2019	8978	B	0	1.57	0.9	0.85	-0.1
F2	19-12-2019	8983	B	0	1.95	0.84	0.69	-0.2
F2	19-12-2019	8979	B	0	0.75	0.53	0.33	-0.5
F2	19-12-2019	8977	B	0	2.12	1.11	1.04	-0.1
F2	19-12-2019	8973	B	0	1.76	0.77	0.61	-0.2
F2	19-12-2019	8968	B	0	0.52	0.56	0.35	-0.5
F2	19-12-2019	8970	B	0	1.37	0.96	0.59	-0.5
F2	19-12-2019	8975	B	0	1.82	0.85	0.77	-0.1
F2	19-12-2019	8976	B	0	1.63	1.05	0.79	-0.3
F2	19-12-2019	8971	B	0	0.68	0.63	0.29	-0.8
F2	19-12-2019	8974	B	0	1.17	0.49	0.45	-0.1
F2	19-12-2019	8966	B	0	1.43	0.75	0.57	-0.3
F2	19-12-2019	8967	B	0	1.65	0.89	0.72	-0.2
F2	19-12-2019	8969	B	0	1.96	1.13	1.13	0
F2	19-12-2019	8972	B	0	1.97	0.98	0.79	-0.2
F2	19-12-2019	8921	B	0	1.65	0.93	0.76	-0.2
F1	19-12-2019	3602	Y	0				
F1	19-12-2019	3989	Y	0				
F1	19-12-2019	3990	Y	0				
F1	19-12-2019	3991	Y	0				
F1	19-12-2019	3992	Y	0				
F1	19-12-2019	3993	Y	0				
F1	19-12-2019	3994	Y	0				
F1	19-12-2019	3995	Y	0				
F1	19-12-2019	3996	Y	0				
F1	19-12-2019	3997	Y	0				
F1	19-12-2019	3999	Y	0				
F1	19-12-2019	4000	Y	0				
F1	19-12-2019	1984	Y	0				
F1	19-12-2019	1478	Y	0				
F1	19-12-2019	1885	Y	0				
F1	19-12-2019	1207	Y	0				
F1	19-12-2019	2076	Y	0				
F1	19-12-2019	2050	Y	0				
F1	19-12-2019	1996	Y	0				
F1	19-12-2019	1566	Y	0				
F1	19-12-2019	10196	Y	0				
F1	19-12-2019	2011	Y	0				
F1	19-12-2019	2504	Y	0				
F1	19-12-2019	2484	Y	0				
F1	19-12-2019	2441	Y	0				
F1	19-12-2019	2461	Y	0				
F1	19-12-2019	3006	Y	0				
F1	19-12-2019	3059	Y	0				
F1	19-12-2019	3087	Y	0				
F1	19-12-2019	3123	Y	0				
F1	19-12-2019	3117	Y	0				
F1	19-12-2019	3020	Y	0				
F1	19-12-2019	3572	Y	0				
F1	19-12-2019	3099	Y	0				

F1	19-12-2019	3049	Y	0
F1	19-12-2019	2950	Y	0
F1	19-12-2019	3558	Y	0
F1	19-12-2019	2492	Y	0
F1	19-12-2019	1970	Y	0
F1	19-12-2019	2576	Y	0
F1	19-12-2019	3617	Y	0
F1	19-12-2019	3016	Y	0
F1	19-12-2019	3032	Y	0
F1	19-12-2019	3131	Y	0
F1	19-12-2019	2564	Y	0
F1	19-12-2019	3142	Y	0
F1	19-12-2019	3544	Y	0
F1	19-12-2019	3056	Y	0
F1	19-12-2019	2539	Y	0
F1	19-12-2019	3608	Y	0
F1	19-12-2019	3446	Y	0
F1	19-12-2019	2530	Y	0
F1	19-12-2019	2354	Y	0
F1	19-12-2019	3560	Y	0
F1	19-12-2019	2480	Y	0
F1	19-12-2019	3057	Y	0
F1	19-12-2019	2542	Y	0
F1	19-12-2019	3063	Y	0
F1	19-12-2019	3046	Y	0
F1	19-12-2019	3581	Y	0
F1	19-12-2019	3086	Y	0
F1	19-12-2019	2459	Y	0
F1	19-12-2019	3546	Y	0
F1	19-12-2019	3019	Y	0
F1	19-12-2019	1943	Y	0
F1	19-12-2019	1975	Y	0
F1	19-12-2019	3961	Y	0
F1	19-12-2019	3025	Y	0
F1	19-12-2019	2485	Y	0
F1	19-12-2019	1957	Y	0
F1	19-12-2019	2440	Y	0
F1	19-12-2019	2051	Y	0
F1	19-12-2019	3074	Y	0
F1	19-12-2019	2960	Y	0
F1	19-12-2019	3910	Y	0
F1	19-12-2019	2434	Y	0
F1	19-12-2019	1324	Y	0
F1	19-12-2019	2972	Y	0
F1	19-12-2019	1500	Y	0
F1	19-12-2019	2980	Y	0
F1	19-12-2019	1959	Y	0
F1	19-12-2019	2490	Y	0
F1	19-12-2019	2489	Y	0
F1	19-12-2019	3958	Y	0
F1	19-12-2019	2436	Y	0

F1	19-12-2019	2986	Y	0
F1	19-12-2019	3101	Y	0
F1	19-12-2019	2472	Y	0
F1	19-12-2019	2007	Y	0
F1	19-12-2019	1954	Y	0
F1	19-12-2019	3964	Y	0
F1	19-12-2019	1484	Y	0
F1	19-12-2019	2449	Y	0
F1	19-12-2019	2349	Y	0
F1	19-12-2019	1475	Y	0
F1	19-12-2019	3072	Y	0
F1	19-12-2019	3955	Y	0
F1	19-12-2019	2426	Y	0
F1	19-12-2019	3957	Y	0
F1	19-12-2019	3953	Y	0
F1	19-12-2019	3023	Y	0
F1	19-12-2019	2432	Y	0
F1	19-12-2019	2355	Y	0
F1	19-12-2019	2466	Y	0
F1	19-12-2019	3956	Y	0
F1	19-12-2019	2381	Y	0
F1	19-12-2019	3547	Y	0
F1	19-12-2019	3551	Y	0
F1	19-12-2019	3013	Y	0
F1	19-12-2019	1151	Y	0
F1	19-12-2019	2973	Y	0
F1	19-12-2019	3549	Y	0
F1	19-12-2019	3954	Y	0
F1	19-12-2019	3034	Y	0
F1	19-12-2019	1438	Y	0
F1	19-12-2019	3553	Y	0
F1	19-12-2019	1966	Y	0
F1	19-12-2019	2997	Y	0
F1	19-12-2019	3550	Y	0
F1	19-12-2019	3959	Y	0
F2	19-12-2019	9013	Y	0
F2	19-12-2019	9011	Y	0
F2	19-12-2019	9012	Y	0
F2	19-12-2019	9020	Y	0
F2	19-12-2019	9018	Y	0
F2	19-12-2019	9015	Y	0
F2	19-12-2019	9009	Y	0
F2	19-12-2019	9017	Y	0
F2	19-12-2019	9010	Y	0
F2	19-12-2019	9019	Y	0
F2	19-12-2019	9016	Y	0
F2	19-12-2019	9014	Y	0
F2	19-12-2019	6964	Y	0
F2	19-12-2019	6532	Y	0
F2	19-12-2019	6902	Y	0
F2	19-12-2019	7521	Y	0

F2	19-12-2019	7485	Y	0
F2	19-12-2019	7880	Y	0
F2	19-12-2019	7453	Y	0
F2	19-12-2019	7018	Y	0
F2	19-12-2019	7399	Y	0
F2	19-12-2019	7911	Y	0
F2	19-12-2019	7901	Y	0
F2	19-12-2019	7886	Y	0
F2	19-12-2019	7423	Y	0
F2	19-12-2019	7425	Y	0
F2	19-12-2019	7732	Y	0
F2	19-12-2019	6533	Y	0
F2	19-12-2019	7456	Y	0
F2	19-12-2019	7885	Y	0
F2	19-12-2019	8485	Y	0
F2	19-12-2019	7424	Y	0
F2	19-12-2019	7361	Y	0
F2	19-12-2019	7844	Y	0
F2	19-12-2019	6945	Y	0
F2	19-12-2019	7920	Y	0
F2	19-12-2019	7883	Y	0
F2	19-12-2019	6257	Y	0
F2	19-12-2019	6962	Y	0
F2	19-12-2019	7388	Y	0
F2	19-12-2019	6983	Y	0
F2	19-12-2019	6931	Y	0
F2	19-12-2019	7503	Y	0
F2	19-12-2019	7887	Y	0
F2	19-12-2019	6924	Y	0
F2	19-12-2019	6892	Y	0
F2	19-12-2019	7513	Y	0
F2	19-12-2019	7409	Y	0
F2	19-12-2019	7982	Y	0
F2	19-12-2019	7413	Y	0
F2	19-12-2019	7909	Y	0
F2	19-12-2019	7450	Y	0
F2	19-12-2019	8501	Y	0
F2	19-12-2019	8516	Y	0
F2	19-12-2019	7861	Y	0
F2	19-12-2019	8467	Y	0
F2	19-12-2019	7951	Y	0
F2	19-12-2019	8490	Y	0
F2	19-12-2019	8498	Y	0
F2	19-12-2019	8574	Y	0
F2	19-12-2019	7831	Y	0
F2	19-12-2019	7858	Y	0
F2	19-12-2019	8513	Y	0
F2	19-12-2019	8439	Y	0
F2	19-12-2019	7912	Y	0
F2	19-12-2019	7819	Y	0
F2	19-12-2019	8434	Y	0

F2	19-12-2019	8483	Y	0
F2	19-12-2019	8423	Y	0
F2	19-12-2019	8507	Y	0
F2	19-12-2019	8430	Y	0
F2	19-12-2019	6916	Y	0
F2	19-12-2019	7868	Y	0
F2	19-12-2019	7888	Y	0
F2	19-12-2019	8450	Y	0
F2	19-12-2019	7846	Y	0
F2	19-12-2019	7345	Y	0
F2	19-12-2019	7408	Y	0
F2	19-12-2019	7877	Y	0
F2	19-12-2019	7894	Y	0
F2	19-12-2019	7904	Y	0
F2	19-12-2019	7827	Y	0
F2	19-12-2019	7931	Y	0
F2	19-12-2019	6918	Y	0
F2	19-12-2019	7836	Y	0
F2	19-12-2019	8503	Y	0
F2	19-12-2019	7825	Y	0
F2	19-12-2019	8461	Y	0
F2	19-12-2019	7443	Y	0
F2	19-12-2019	7404	Y	0
F2	19-12-2019	8419	Y	0
F2	19-12-2019	7873	Y	0
F2	19-12-2019	7403	Y	0
F2	19-12-2019	7853	Y	0
F2	19-12-2019	7770	Y	0
F2	19-12-2019	7740	Y	0
F2	19-12-2019	8412	Y	0
F2	19-12-2019	7899	Y	0
F2	19-12-2019	7856	Y	0
F2	19-12-2019	7448	Y	0
F2	19-12-2019	8489	Y	0
F2	19-12-2019	8592	Y	0
F2	19-12-2019	8982	Y	0
F2	19-12-2019	8981	Y	0
F2	19-12-2019	8978	Y	0
F2	19-12-2019	8983	Y	0
F2	19-12-2019	8979	Y	0
F2	19-12-2019	8977	Y	0
F2	19-12-2019	8973	Y	0
F2	19-12-2019	8968	Y	0
F2	19-12-2019	8970	Y	0
F2	19-12-2019	8975	Y	0
F2	19-12-2019	8976	Y	0
F2	19-12-2019	8971	Y	0
F2	19-12-2019	8974	Y	0
F2	19-12-2019	8966	Y	0
F2	19-12-2019	8967	Y	0
F2	19-12-2019	8969	Y	0

F2	19-12-2019	8972	Y	0	
F2	19-12-2019	8921	Y	0	
N1	03-12-2019	4	S	0	
N1	03-12-2019	3	S	0	
N1	03-12-2019	5	S	0	
N1	03-12-2019	17	S	0	
N1	03-12-2019	16	S	0	
N1	03-12-2019	15	S	0	
N1	03-12-2019	14	S	0	
N1	03-12-2019	13	S	38.96	PRRSV1
N1	03-12-2019	10	S	31.86	PRRSV2
N1	03-12-2019	0	S	31.16	PRRSV2
N2	03-12-2019	10	S	0	
N2	03-12-2019	9	S	0	
N2	03-12-2019	8	S	0	
N2	03-12-2019	7	S	0	
N2	03-12-2019	6	S	0	
N2	03-12-2019	5	S	0	
N2	03-12-2019	4	S	0	
N2	03-12-2019	3	S	32.14	PRRSV2
N2	03-12-2019	2	S	0	
N2	03-12-2019	1	S	0	
N1	19-12-2019	4	S	0	
N1	19-12-2019	3	S	0	
N1	19-12-2019	5	S	0	
N1	19-12-2019	17	S	0	
N1	19-12-2019	16	S	0	
N1	19-12-2019	15	S	0	
N1	19-12-2019	14	S	0	
N1	19-12-2019	13	S	0	
N1	19-12-2019	8	S	0	
N1	19-12-2019	9	S	0	
N1	19-12-2019	10	S	0	
N1	19-12-2019	11	S	0	
N1	19-12-2019	12	S	0	
N2	19-12-2019	10	S	0	
N2	19-12-2019	9	S	0	
N2	19-12-2019	8	S	0	
N2	19-12-2019	7	S	0	
N2	19-12-2019	6	S	37.81	PRRSV2
N2	19-12-2019	3	S	0	
N2	19-12-2019	2	S	0	
N2	19-12-2019	1	S	0	
N1	25-02-2020	3	S	0	
N1	25-02-2020	4	S	0	
N1	25-02-2020	5	S	0	
N1	25-02-2020	8	S	35.39	PRRSV2
N1	25-02-2020	9	S	0	
N1	25-02-2020	10	S	0	
N1	25-02-2020	11	S	0	
N1	25-02-2020	12	S	0	

N1	25-02-2020	13	S	29.46	PRRSV2
N1	25-02-2020	14	S	28.73	PRRSV2
N1	25-02-2020	15	S	29.92	PRRSV2
N1	25-02-2020	16	S	0	
N1	25-02-2020	17	S	0	
N2	25-02-2020	1	S	0	
N2	25-02-2020	2	S	0	
N2	25-02-2020	3	S	0	
N2	25-02-2020	9	S	33.83	PRRSV1
G2	25-02-2020	10	S	33.28	PRRSV1

Table S2 – Dichotomous results of serological assays.

Table S2. Dichotomous PRRSV-1 S/P antibody results in Idexx ELISA and MFIA before and after PRRSV-1 MLV mass vaccination in the study of two herds.

F1 - before vaccination			
	Idexx ELISA positive	Idexx ELISA negative	Sum
MFIA-positive	90	3	93
MFIA-negative	4	15	19
Sum	94	18	112
F1 - after vaccination			
	Idexx ELISA positive	Idexx ELISA negative	Sum
MFIA-positive	96	1	97
MFIA-negative	2	3	5
Sum	98	4	102
F2 - before vaccination			
	Idexx ELISA positive	Idexx ELISA negative	Sum
MFIA-positive	101	2	103
MFIA-negative	4	7	11
Sum	105	9	114
F2 - after vaccination			
	Idexx ELISA positive	Idexx ELISA negative	Sum
MFIA-positive	107	1	108
MFIA-negative	0	1	1
Sum	107	2	109
F1 + F2 - before vaccination			
	Idexx ELISA positive	Idexx ELISA negative	Sum
MFIA-positive	191	5	196
MFIA-negative	8	22	30
Sum	199	27	226
F1 + F2 - after vaccination			
	Idexx ELISA positive	Idexx ELISA negative	Sum
MFIA-positive	203	2	205
MFIA-negative	2	4	6
Sum	205	6	211

Table S3 – Spearman's Correlation

Table S3. Results of the spearman correlation test between results of PRRSV-1 S/P-value in both Idexx ELISA (x-axis) and MFIA (y-axis) before (−2DPV) and after PRRSV-1 MLV mass vaccination (WPV2) in farm 1 (F1) and farm 2 (F2), and in total for both herds (both) in the study. Spearman's correlation coefficients for the monotonic relationship (r_s), P-value (p) and 95% confidence interval (95% CI) are presented.

**Spearman's correlation**									
	F1			F2			Both		
Time	r_s	95% CI	*p*	r_s	95% CI	*p*	r_s	95% CI	*p*
−2DPV	0.88	0.83-0.92	<0.0001	0.88	0.83-0.92	<0.0001	0.89	0.85-0.91	<0.0001
WPV2	0.81	0.73-0.87	<0.0001	0.86	0.80-0.90	<0.0001	0.83	0.79-0.87	<0.0001
Total	0.86	0.83-0.90	<0.0001	0.92	0.89-0.93	<0.0001	0.89	0.87-0.91	<0.0001

Table S4 – Cohen's Kappa agreement
Cohen's kappa agreement coefficient (κ) between PRRSV-1 S/P antibody results in Idexx ELISA and MFIA before (−2DPV) and after PRRSV-1 MLV mass vaccination (WPV2) and in total for both serologic assays in the study of two herds (F1 and F2). Furthermore, standard error (*SE*), 95% confidence intervals (95% CI), observed agreements chance in numerical values and proportion and observed agreements by chance in numerical values and proportion.

**F1**					
	κ	*SE*	95% CI	Observed agreements	… by chance
−2DPV	0.77	0.08	0.61-0.93	105 (93.75%)	81.1 (72.42%)
WPV2	0.65	0.18	0.28-1.00	99 (97.06%)	93.4 (91.56%)
Total	0.76	0.07	0.61-0.90	204 (95.33%)	172.9 (80.81%)

**F2**					
	κ	*SE*	95% CI	Observed agreements	… by chance
−2DPV	0.67	0.13	0.43-0.92	108 (94.74%)	95.7 (83.98%)
WPV2	0.66	0.32	0.04-1.00	108 (99.08%)	106.0 (97.28%)
Total	0.68	0.11	0.46-0.90	216 (96.87%)	201.2 (90.22%)
